# Molecular factors modulating the anti-inflammatory activity of IVIg in ITP

**DOI:** 10.1186/1479-5876-10-S3-P15

**Published:** 2012-11-28

**Authors:** Inessa Schwab, Falk Nimmerjahn

**Affiliations:** 1 Dept. of Biology, University of Erlangen-Nürnberg, Erlangen, Germany

## Background

Intravenous Immunoglobulin G (IVIg) therapy is widely used to treat autoimmune diseases. A variety of mechanisms have been suggested to be responsible for the anti-inflammatory activity of IVIg. Among these, terminal sialic acid residues in the sugar moiety of the IgG Fc-fragment have been shown to be critical for its anti-inflammatory activity in a model of serum transfer arthritis. Moreover, splenic resident cells expressing specific-ICAM3 grabbing nonintegrin-related1 (SIGNR1) were shown to have the capacity to bind antibodies rich in sialic acid residues, suggesting a role of the spleen in this immunomodulatory pathway. Recently, B cells and the sialic acid-binding protein CD22 were suggested to be involved in the IVIg-mediated anti-inflammatory process.

## Material and methods

To induce a murine model of thrombocytopenia (ITP), mice were injected with the specific anti-platelet antibody 6A6-IgG2a. Mice were either pretreated with IVIg or PBS two hours prior to platelet depletion. Splenectomized mice and several mouse strains lacking B cells, CD22 or SIGNR1 were used for experiments. Flow cytometric analysis revealed *in vivo* binding of human IgG-molecules to different cell populations in peripheral blood of IVIg treated mice. Deglycosylation of IVIg preparations was achieved by enzymatic cleavage with neuraminidase or PNGaseF.

## Results

Mice treated with de-glycosylated IVIg lacking either the whole sugar moiety or the terminal sialic acid residue were not protected from ITP. We demonstrated that IVIg activity was still functional in splenectomized mice but could be blocked with a specific anti-SIGNR1 antibody. Despite the capacity of IVIg to recognize sialic acid rich IgG via CD22 on B cells, neither B cells nor CD22 were involved in IVIg dependent suppression in autoantibody induced ITP. Binding efficiency of IVIg to myeloid cells and leukocytes was neither altered in mice lacking B cells or CD22 nor in mice treated with de-syalylated IVIg.

## Conclusions

These results suggest a spleen-independent but sialic acid- and SIGNR1-dependent mechanism responsible for IVIg effects in ITP. Moreover, neither B cells nor CD22 are critical for the anti-inflammatory activity of IVIg.

